# Emerging Therapies in CLL in the Era of Precision Medicine

**DOI:** 10.3390/cancers15051583

**Published:** 2023-03-03

**Authors:** Prajish Iyer, Lili Wang

**Affiliations:** 1 Department of Systems Biology, Beckman Research Institute, City of Hope National Comprehensive Cancer Center, Monrovia, CA 91007, USA; 2 Toni Stephenson Lymphoma Center, Beckman Research Institute, City of Hope National Comprehensive Cancer Center, Duarte, CA 91016, USA

**Keywords:** CLL, emerging therapies, metabolism, splicing, whole-exome, transcriptome

## Abstract

**Simple Summary:**

Despite being a slow-proliferating disease, chronic lymphocytic leukemia (CLL) is an incurable and frequently reoccurring adult leukemia. Although large-scale genome-wide next-generation sequencing studies have provided insights into CLL’s transcriptome and mutational landscape, the molecular mechanisms underlying disease progression remain incompletely understood. Over time, the treatment landscape in CLL has shifted from chemoimmunotherapies (CIT) to targeted therapies, but resistance mechanisms have emerged, leading to progression such as Richter’s transformation (RT). As a result, there remains an unmet clinical need to identify new therapeutic strategies. In our review article, we aim to evaluate the past and current state of CLL treatment in both frontline and relapsed/refractory settings and also explore mitochondrial reprogramming, metabolic alterations, and RNA splicing as potential novel therapeutic targets.

**Abstract:**

Over the past decade, the treatment landscape of CLL has vastly changed from the conventional FC (fludarabine and cyclophosphamide) and FCR (FC with rituximab) chemotherapies to targeted therapies, including inhibitors of Bruton tyrosine kinase (BTK) and phosphatidylinositol 3-kinase (PI3K) as well as inhibitors of BCL2. These treatment options dramatically improved clinical outcomes; however, not all patients respond well to these therapies, especially high-risk patients. Clinical trials of immune checkpoint inhibitors (PD-1, CTLA4) and chimeric antigen receptor T (CAR T) or NK (CAR NK) cell treatment have shown some efficacy; still, long-term outcomes and safety issues have yet to be determined. CLL remains an incurable disease. Thus, there are unmet needs to discover new molecular pathways with targeted or combination therapies to cure the disease. Large-scale genome-wide whole-exome and whole-genome sequencing studies have discovered genetic alterations associated with disease progression, refined the prognostic markers in CLL, identified mutations underlying drug resistance, and pointed out critical targets to treat the disease. More recently, transcriptome and proteome landscape characterization further stratified the disease and revealed novel therapeutic targets in CLL. In this review, we briefly summarize the past and present available single or combination therapies, focusing on potential emerging therapies to address the unmet clinical needs in CLL.

## 1. Introduction

CLL is one of the most common forms of adult leukemia in the western world, characterized by the accumulation of CD19^+^CD5^+^ cells in bone marrow, lymph nodes, the spleen, and peripheral blood [1]. Typically, CLL occurs in the older age group with a median age at diagnosis of 72. In 2020, it was estimated that 21,040 new cases and 4060 deaths in the USA per year [2]. Despite all the treatment advancements in the past decade, CLL remains incurable. Approximately 10% of patients progress to an aggressive form of lymphoma called RT, and at least 20% develop chemorefractory disease or resistance to targeted therapies [3]. Thus, novel treatment options are still needed to cure this disease.

In the past decade, large-scale genome-wide whole-exome and whole-genome sequencing studies of primary CLL samples have revealed the mutational landscape of CLL and its vast genetic heterogeneity [4,5,6]. Integration of somatic mutations and clinical annotation enabled the identification of genetic drivers and the improvement of the prognostication of CLL patients. Recurrent alterations have been identified in genes related to significant pathways such as RNA splicing and metabolism (*SF3B1*, *U1*, *RPS15*, *DDX3X*), DNA damage (*ATM*, *TP53*), MAPK-ERK (*KRAS*, *BRAF*, *NRAS*), B-cell receptor and Toll-like receptor signaling (*MYD88*, *PAX5*, *BCOR*, *IKZF3*), the cell cycle (*CDKN1B*, *CDKN2A*), NF-KB signaling (*BIRC3*, *TRAF2*, *TRAF3*), and chromatin modification (*CHD2*, *SETD2*, *KMT2D*, and *ASXL1*) [4,7,8,9,10,11]. Genetic heterogeneity functions as a fuel for clonal evolution and is implicated in disease progression and poor prognosis [12]. More in-depth studies uncovered that genetic heterogeneity is influenced by the cell of origin; for example, some mutations appear majorly in immunoglobulin heavy chain variable region gene (IGHV) unmutated CLL (U-CLL) (*U1snRNA*, *NOTCH1*, *POT1*), whereas others present predominantly in IGHV mutated CLL (M-CLL)(*MYD88*, *PAX5)*, in the presence of sub-clonal mutations that are acquired during the disease evolution, according to the age of CLL patients (young patients have *MYD88* mutations) [13,14], or according to the course of the disease (MYC amplification is acquired during transformation to aggressive lymphoma). Identification of mutations and copy number variations on independent cohorts of patients undergoing targeted therapies will unravel the predictive value of CLL management and develop personalized treatments to combat disease resistance and improve progression-free survival (PFS) outcomes.

Studies of CLL biology have also revealed novel aspects of this disease. Splicing factor mutations were identified in ~20% of CLL samples, and splicing dysregulation is reviewed as a characteristic feature of CLL [11,15,16]. Targeting of RNA splicing dysregulation was explored recently [16]. Metabolic reprogramming is a hallmark of cancer and underlies disease progression and relapse. In the past few years, various studies have uncovered glucose, lipid, and glutamine metabolic dependencies in CLL samples [17,18,19]. However, the exploitation of metabolic dependencies in clinical settings is still minimal. This review summarizes past, present, and emerging therapies to combat CLL in frontline and relapsed settings.

## 2. Chemoimmunotherapy

### 2.1. Fludarabine, Cyclophosphamide, and Rituximab (FCR)

Observation without cytotoxic therapy is usually the approach for asymptomatic patients in earlier stages of CLL (Rai stage 0), while therapy is recommended for those with symptomatic CLL [20]. For over a decade, chlorambucil, a purine analog, has been the preferred treatment for CLL patients. From 1997 to 2006, the German CLL study group conducted several trials to improve the survival of CLL patients [21,22,23]. The CLL5 trial demonstrated that older patients did not benefit from first-line therapy with fludarabine, as their median overall survival (OS) was 46 months, whereas it was 64 months with chlorambucil. Nonetheless, fludarabine is more effective than chlorambucil [23]. The CLL4 trial revealed that combining fludarabine with cyclophosphamide enhanced the quality and duration of response in younger patients under the age of 65 when compared to fludarabine alone [21]. The FCR300 trial (fludarabine-cyclophosphamide combined with the CD20-monoclonal antibody, rituximab) showed an overall response rate (ORR) of 95% with a complete remission (CR) of 72% and a PFS of 6 years [24]. Long-term outcomes from the FCR300 trial showed that one-half of the patients who had mutated IGHV and received FCR achieved negative status for minimal residual disease (MRD) with a PFS rate of 79.8 at 12.8 years [20]. Overall, in patients with or without mutated IGHV, the PFS rate at 12.8 years was 53% and 8%, respectively [25]. In phase III of the ECOG-ACRIN 1912 clinical trial, 529 treatment-naïve CLL patients aged ≤70 years were randomly assigned to receive ibrutinib-rituximab (IR)/six cycles of FCR [26]. With a median follow-up of 70 months, the results showed superior PFS with IR compared to FCR in patients with mutated IGHV (HR, 0.27; 95% CI, 0.11–0.62). Of note, ibrutinib treatment was mainly discontinued in one out of five patients due to grade three adverse events, and 60% of patients were randomized to receive ibrutinib-based therapy with approximately six years of follow-up [26]. FCR is also associated with side effects such as myelosuppression and infections. As a result, FCR therapy is not very well-tolerated in older patients aged >65 years. A phase III CLL10 trial showed that bendamustine and rituximab (BR) are tolerable in older patients with comparable efficacy. The results showed that FCR was superior to BR in terms of PFS at 55.2 months in the FCR arm vs. 41.7 months in the BR arm; however, patients > 65 years had increased infections and cytopenias in the FCR arm [27].

### 2.2. Monoclonal Antibody Treatment in CLL

For over a decade, monoclonal antibodies, such as CD20-targeting rituximab and ofatumumab or CD52-targeting alemtuzumab, have been available for CLL patients. Ofatumumab has been shown to bind a specific epitope in CD20 [28], improving complement-dependent cytotoxicity over rituximab. In the PROLONG trial, single-agent ofatumumab improved the PFS and remission in relapsed patients when given maintenance therapy compared to the current standard of care (observation) [29]. Combining ofatumumab with FC or pentostatin and cyclophosphamide has shown equivalent efficacy to FCR [30,31]. Another monoclonal CD52-targeting antibody, alemtuzumab, has demonstrated superiority over chlorambucil in the frontline setting in CAM 307 trials [32]. A combination of alemtuzumab with FCR was tested in 60 high-risk CLL patients as a frontline treatment and showed an OR of 92% of CR 3 grade and grade 3–4 myelotoxicity [33]. Due to significant toxicities such as immunosuppression, cytokine storm, opportunistic infections, and neutropenia, alemtuzumab has limited use in CLL. Recently, a novel monoclonal CD20 antibody called obinutuzumab, humanized glycoengineered (lack of sugar moiety in the Fc region) antibody, has been added to the armamentarium [34]. In the GAUGUIN monotherapy trial, obinutuzumab (Obi) demonstrated an ORR of 62% in phase I and 30% in phase II [34]. In the phase III CLL11 trial conducted by GCLLSG, which involved 781 previously untreated patients with a cumulative illness rating scale score of >6, patients were divided into three arms: obinutuzumab + chlorambucil (Obi-Chl, arm I) compared to rituximab + chlorambucil (Rix-Chl, arm II) and chlorambucil alone (arm III). The median PFS was 26.7 months in arm I compared to 11.1 months in arm III and 16.3 months in arm II (*p* < 0.001). Overall, there was an improvement in ORR, PFS (*p* < 0.001), and OS (*p* = 0.002) in arm I as compared to arm III. Additionally, there was an improvement in PFS (*p* > 0.001) and CR rates (20.7% vs. 7%) when comparing arm I with arm II. Obi has improved efficacy in older patients with coexisting conditions [35]. Based on the results of the CLL11 trial, Obi has been approved by the FDA with chlorambucil for treating previously untreated patients with comorbidities. The GAIA (CLL13) study assessed the effectiveness and safety of three frontline treatments consisting of venetoclax (BCL2 inhibitor) combined with CD20 antibody compared to standard CIT for patients with CLL who were fit and without the *TP53* mutation/deletion. The study followed patients for a median of 38.8 months and found that the median PFS was not reached in patients treated with venetoclax (Ven), obinutuzumab (Obi), and ibrutinib (Ven-Obi-Ibr, HR = 0.32) or Ven and Obi (Ven-Obi, HR = 0.42). By contrast, the median PFS for standard chemotherapy was 52 months. The combination of Ven and rituximab had a similar PFS to CIT. Ven-Obi-Ibr reduced the risk of CLL progression by 68%, and Ven-Obi reduced the risk by 58%. The rates of three-year PFS were 90.5% and 87.7% for Ven-Obi-Ibr and Ven-Obi, respectively. The study also found that patients treated with venetoclax-based regimens had higher rates of MRD negativity. However, overall survival rates were similar across all treatment arms. The CLL14 randomized phase III trial evaluated the combination of Obi with Ven, particularly in elderly patients with coexisting conditions who cannot tolerate intensive CIT such as FCR [36]. About 432 patients were enrolled, and patients were randomized to either 12 cycles of chlorambucil/obinutuzumab (Clb-Obi) or 12 cycles of Ven-Obi, and the main objective was improving PFS. Overall, patients treated with Ven-Obi showed longer PFS compared to those treated with Clb-Obi and had undetectable minimal residual disease (uMRD) levels of 76% at the end of treatment. The adverse events were minimal in both arms, and patients treated with Ven-Obi demonstrated improved global health status, insomnia, fatigue, and quality of life [36].

## 3. Targeted Therapies

### 3.1. Targeting B-Cell Receptor (BCR) Signaling in CLL

**Figure 1:**
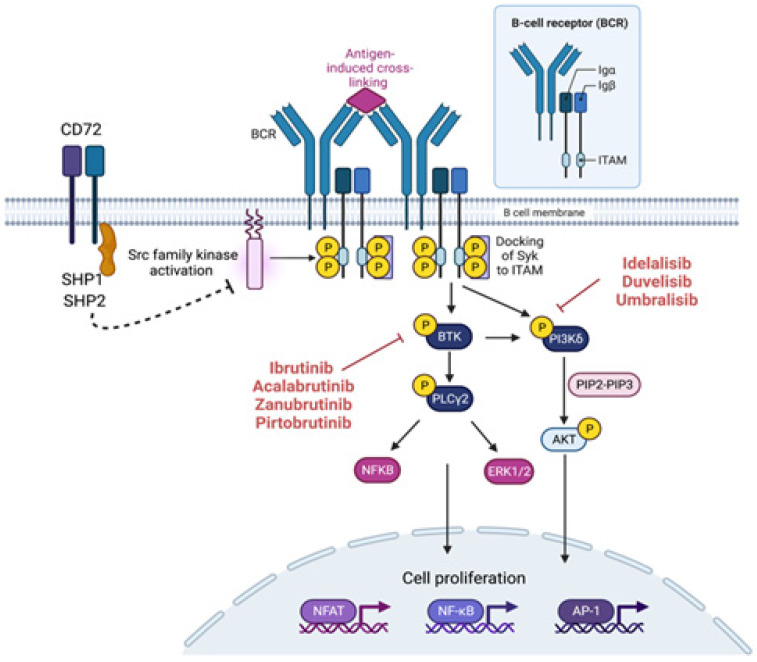
BCR signaling and targeting agents for BCR signaling. Inhibitors of the BTK pathway (red line: left) and inhibitors of the PI3-kinase pathway (red line: right). SHP-1 modulates B-cell function by dephosphorylating various receptors, and downstream molecules (Src kinases-Pink) are recruited to the tyrosine phosphorylated motif of these receptors (dotted line). Created with Biorender.com.

The BCR is composed of a surface immunoglobulin (Ig) molecule non-covalently associated with an Ig-α and Ig-β (CD79a/CD79b) (Figure 1). In normal B-cells, antigenic stimulation leads to signalosome formation, a complex of scaffold proteins and kinases tethered at the plasma membrane at the sites of sIg activation [37]. The antigenic binding leads to the phosphorylation of SRC family kinase LYN, which is further followed by a series of kinases such as SYK, which further recruit B-cell linker protein (BLNK) and other adaptor molecules. This signalosome complex activates phospholipase C-γ2 (PLC-γ2), and Ras. Ras binds to and leads to the activation of Raf, which activates ERK (Extracellular Regulated Kinase). PLC-γ2 activation releases intracellular calcium, which activates PKC (Protein Kinase C). PKC activation leads to subsequent activation of various kinases—MAPK (Mitogen-activated Protein Kinases), c-JUN NH_2_-terminal kinase (JNK), and p38 MAPK and transcription factors such as MYC and NF-κB. BCR is negatively regulated by molecules such as CD22, CD5, CD72, and FcγRIIB that control the duration and intensity of the signaling [38]. These negative regulators contain immunoreceptor tyrosine-based inhibitory motifs [37,39].

Although CLL cells express low levels of surface immunoglobulin [40,41], these cells were found to be driven by antigen-independent autonomous signaling for growth and proliferation, which is dependent on the unique heavy-chain complementarity-determining region (CDR) and an internal epitope of the BCR [42]. It is well-known that a striking aspect of CLL is that the immunoglobin heavy chain (IgVH) and immunoglobin light chain (IgVL) have a very limited repertoire with similar gene rearrangements. Approximately one-third of CLL patients carry quasi-identical BCR sequences that can be classified into stereotyped BCR subsets based on CDR structure. These findings support the idea that tonic BCR signaling is a critical pathogenic mechanism driving CLL [43]. In addition, a strong correlation between BCR signaling and IGHV mutation status was observed [44]. Strong down-modulation of BCR signaling is observed in M-CLL due to a lack of sIgM expression. Less dramatic reduction of sIgM in U-CLL leads to partial activation of downstream pathways. In addition to IgM modulation, other factors also contribute to BCR signaling. ZAP70 expression correlates with BCR signaling, and its overexpression augments BCR signaling capacity [45]. In CLL, ZAP70 overexpression is associated with poor clinical outcomes and expresses unmutated IgHV [46]. Additional evidence suggests that ZAP70 may modulate cell migration-associated pathways. CD38 is another prognostic indicator whose expression correlates with sIgM signaling capacity [47]. Reports indicate that CD38–CD31 interactions contribute to cell migration and homing, enhancing CLL survival via inducing BCL2 and BCL X_L_ [48,49]. Overall, BCR signaling is essential for CLL pathogenesis; thus, targeting this pathway is vital for treating CLL patients.

### 3.2. Dasatinib and Ibrutinib

Targeted tyrosine kinase inhibitors have been of particular interest in treating CLL due to the success of using tyrosine kinase inhibitors in CML. Dasatinib, which has been used to treat CML and Ph^+^ ALL acute lymphoblastic leukemia (ALL), can also inhibit the Src family of kinases, including Lyn, which is often dysregulated in CLL B-cells. Dasatinib induces apoptosis in CLL B-cells in vitro, with U-CLL cells being more sensitive [50], and has also been shown to sensitize tumor cells to chlorambucil and fludarabine to overcome CD40-mediated drug resistance in vitro [51]. A phase II study of dasatinib in high-risk relapsed or refractory CLL patients has shown a partial response with side effects of myelosuppression in two-thirds of the cases [52]. A recent in vitro study with 53 CLL patient samples treated with dasatinib showed that 17.7% of samples were apoptotic, indicating that dasatinib has anti-leukemic effects [53]; however, clinical use of dasatinib has been unclear.

Ibrutinib, a covalent inhibitor for Bruton’s tyrosine kinase (BTK), was approved by the FDA as one of the first inhibitors for treating relapsed or refractory (R/R) CLL after showing positive results in the RESONATE trial, a randomized phase III trial that compared ibrutinib with a single agent ofatumumab in R/R CLL patients [54]. The 18-month PFS was similar regardless of the genetic mutations, including *NOTCH1*, *BIRC3*, and *ATM*, or other factors such as del(17p) and del(11q) [54]. In addition, a phase Ib/II single-arm trial compared ibrutinib with single-agent PCYC-1102 treatment, where patients with R/R and TN settings were treated with ibrutinib as a single agent. A pooled analysis from the RESONATE and PCYC-1102 trials showed that patients who received ibrutinib and no prior treatment or bulky disease (lymph node ≥ 5 cm) achieved a CR. Another RESONATE-2 trial compared ibrutinib with chlorambucil in 269 treatment-naïve older (65+) patients with CLL/SLL, where ibrutinib showed large improvements at ORR [55]. RESONATE-17 was a single-arm study of ibrutinib in 144 R/R CLL or SLL del(17p). The results showed that the 24-month PFS, OS, and ORR were 63%, 75%, and 83%, respectively, after a median follow-up of 11.5 months [56]. Notably, complex karyotype (CK) may be a strong predictor of inferior outcomes compared to del(17p) among patients with R/R CLL in the setting of ibrutinib therapy. The MD Anderson Cancer Center analyzed 88 patients with R/R CLL who received ibrutinib-based therapies between 2010 and 2013. Investigators found that only fludarabine refractoriness and complex karyotype were statistically significant prognostic factors associated with lower OS, with an HR of 6.9 and 5.9, respectively. Interestingly, del(17p) was not associated with a decreased OS [57].

In light of the success of ibrutinib, second-generation BTK inhibitors (BTKi) such as acalabrutinib and zanubrutinib have been developed aiming to reduce grade 2–4 adverse events, bleeding, and cardiac toxicities. In the ASCEND trial, a phase III trial focused on cytogenetic high-risk R/R patients, acalabrutinib demonstrated superior PFS compared to CIT and the PI3Kδ inhibitor idelalisib [58]. Furthermore, the ELEVATE-TN trial examined first-line treatment in elderly CLL patients with comorbidities, which showed that single-agent acalabrutinib or in combination with the anti-CD20 mAb obinutuzumab can prolong PFS [59]. In a recent ELEVATE-RR phase III-based clinical trial dedicated to R/R CLL patients, acalabrutinib treatment was non-inferior in terms of PFS (38.6 months) compared to ibrutinib (38.4 months PFS) and had an improved safety profile with fewer AF events [60]. Zanubrutinib has demonstrated an ORR of 85% in phase II clinical trials with treatment-naïve (TN) and R/R CLL with *TP53* mutations [61]. In a randomized phase III controlled clinical trial, zanubrutinib single treatment was compared with ibrutinib and zanubrutinib in R/R CLL patients [62]. Zanubrutinib has shown a superior response rate and improved PFS with a lower rate of atrial fibrillation than ibrutinib [63]. Similar to other kinase inhibitors, resistance was developed by either acquired mutations in the BTK domain, such as mutations in the drug binding site (BTK Cys481) [64] PLCG2 mutations [65], or alterations, such as del(8p) and additional mutations in *EP300*, *MLL2*, and *EIF2A*. To overcome the BTKi resistance, pirtobrutinib has been developed as an orally available, highly selective reversible BTKi with equal potency against wildtype and Cys481 mutated BTK. It has shown to have an overall good safety profile with an ORR of 62% in phase I/II studies of R/R CLL patients [66]. Mechanisms for non-genetic events-related resistance were under investigation in CLL and other B-cell malignancies with candidate treatment options proposed [67,68].

### 3.3. PI3 Kinase Inhibitors

The p110δ isoform of PI3K is responsible for transducing downstream signals of BCR signaling. Although idelalisib has been approved, many concerns exist regarding the toxicity and modest outcomes associated with treatment. In particular, compared to the relatively safer ibrutinib, the use of idelalisib in patients with CLL has been limited. Thus, it is critical to exercise a high degree of vigilance for these adverse events in patients receiving idelalisib.

#### 3.3.1. Idelalisib (p110-PI3Kδ Inhibitor)

Idelalisib (IDEL) is a selective PI3Kδ inhibitor that promotes the apoptosis of B-cells. Activating the BCR signaling increases the calcium, which increases the diacylglycerol and IP3, thus activating the PI3 kinase pathway. Hence, treatment of CLL cells with PI3Kδ inhibitors causes a reduction in lymph node size and concomitant enhanced lymphocytosis, which can be mitigated using rituximab. In phase III, a randomized, double-blind, placebo-controlled trial (220 high-risk R/R CLL patients) of idelalisib + rituximab (IDEL-RIX) versus a placebo + rituximab, the IDEL-RIX arm had significantly improved ORR compared to the placebo arm (81% vs. 13% *p* < 0.001), as well as OS (92% vs. 80% at 12 months *p* = 0.02), irrespective of the presence of poor prognostic factors including del17p [69]. In this phase III trial, the adverse events were similar in both the idelalisib and placebo groups; in the idelalisib group, there were five common adverse events like fatigue, pyrexia, nausea, chills, and diarrhea. Grade 3–4 elevation of hepatic transaminases occurred more in the idelalisib group. Gastrointestinal and skin disorders led to discontinuation in the idelalisib group [70]. In another phase II trial involving 64 patients, IDEL-RIX showed an ORR of 97% with a CR of 19%. Despite its efficacies, grade 2–3 AEs (adverse events) such as transaminitis, rash, and diarrhea have been reported [71]. All these AEs are managed with a drug hold; hence, the US FDA approved idelalisib in combination with rituximab for high-risk R/R CLL patients.

#### 3.3.2. Duvelisib

Duvelisib is an inhibitor that targets two forms of the phosphatidyl 3-kinase (PI3K) enzyme, specifically the PI3Kδ and γ forms, and has been used to manage high-risk CLL patients. In September 2018, the US FDA approved duvelisib for treating R/R CLL patients after having undergone two prior lines of therapy. In a phase I dose-escalation trial involving 52 R/R CLL patients and 15 older previously untreated patients with CLL, duvelisib showed an ORR of 48% with 89% nodal responses [72]. Currently, duvelisib is undergoing testing in a phase III trial in comparison with ofatumumab for relapsed CLL patients. In a randomized phase III DUO trial, duvelisib was compared with ofatumumab monotherapy in patients with R/R CLL. Duvelisib showed a significantly higher ORR (74%) than the ofatumumab arm (45%; *p* = 0.0001). The median PFS was 13.3 months in the duvelisib arm versus 9.9 months in ofatumumab, respectively, among all patients. The median PFS of del(17p) or patients with the *TP53* mutation was 12.7 months on treatment with duvelisib, while the median survival of patients without such lesions was not reported [73]. The most common adverse events are diarrhea, neutropenia, pyrexia, nausea, anemia, and opportunistic infections such as *P jirovecii* pneumonia in patients who did not receive prophylactic treatment. These data indicate that duvelisib may be effective for R/R CLL patients.

#### 3.3.3. Umbralisib

Umbralisib is a novel PI3K inhibitor with efficacy similar to idelalisib is umbralisib, which has been shown to have low toxicity in R/R CLL patients. In the phase I–II trials comprising a triplet combination of umbralisib with ublituximab plus venetoclax, the ORR was 90% with a CR of 29% and a two-year PFS of 90%. In the peripheral blood and bone marrow, 58% of patients had undetectable MRD [74]. Due to findings from the UNITY-CLL clinical trial (NCT02612311) continuing to show a possible increased risk of death in patients receiving umbralisib, TG Therapeutics withdrew it from the market in June 2022.

### 3.4. Targeting BCL2

B-cell leukemia/lymphoma-2 (BCL2) is a protein often overexpressed in CLL patients and plays a crucial role in regulating the apoptotic pathway. Venetoclax is a highly selective, second-generation small-molecule inhibitor of BCL2 that can effectively shift the balance of proteins in CLL cells towards apoptosis. In the phase I clinical trial that included 116 high-risk R/R CLL patients, venetoclax achieved an ORR of 79%, with 20% of patients experiencing a CR. Notably, patients with del(17p) and those who were refractory to fludarabine achieved even higher ORRs of 82% and 89%, respectively. The most common grade 3–4 adverse event reported was neutropenia, in 41% of patients [75]. Currently, there are several combination trials underway involving venetoclax. In one study that examined the combination of venetoclax and rituximab (Ven-Rix) in high-risk R/R CLL patients, approximately half of the patients achieved CR, with 57% achieving uMRD activity [76]. The estimated two-year PFS was 82% (95% CI 66–91); however, two patients progressed after 24 months of therapy [76]. The phase III MURANO trial compared a combination of Ven-Rix versus bendamustine/rituximab (BR) in R/R CLL patients. The two-year PFS rate was 84.9% and 36.3% in the Ven-Rix arm and the BR arm, respectively, with a median PFS significantly higher in the Ven-Rix arm [77]. Tumor lysis syndrome (TLS) is one of the most common adverse events associated with venetoclax treatment. Hence, appropriate patient selection, strategies to reduce TLS risk, and following the standard ramp-up will be critical for successful treatment with low TLS risk. Preclinical studies investigating the synergy of BTK and BCL2 inhibitors and single-center studies examining the effectiveness of ibrutinib in combination with venetoclax with or without obinutuzumab have shown promising results in CLL treatment. A phase II international study named CAPTIVATE (NCT02910583) examined the use of fixed-duration treatment with ibrutinib plus venetoclax (Ibr-Ven) in patients aged ≤70 years who had not received any prior treatment for CLL [78]. Despite a median follow-up of only 27.9 months, the results seen in this trial are remarkable. The treatment regimen demonstrated exceptional efficacy with a 56% CR, and with 76% and 62% of patients achieving uMRD in the blood and bone marrow. At the 24-month mark, 95% of patients were alive and free of progression. Notably, patients with high genomic risk diseases such as *TP53* abnormalities had excellent outcomes. Additionally, patients with unmutated IGHV showed a trend toward achieving uMRD [78]. A phase III trial named GLOW (*n* = 211) evaluated the efficacy and safety of Ibr-Ven in older patients and/or those with comorbidities with previously untreated CLL. After a median follow-up of 46 months, the Ibr-Ven treatment was found to reduce the risk of disease progression or death by 79% compared to chlorambucil with Obi (Clb-Obi) (Hazard Ratio (HR) 0.214; 95% Confidence Interval (CI), 0.138–0.334; *p* < 0.0001). This marks the first instance of a fixed-duration novel combination demonstrating an OS advantage compared to Clb-Obi as a first-line treatment for CLL (HR 0.487; 95% CI, 0.262–0.907; nominal *p* = 0.0205) with the Ibr-Ven treatment given once daily orally. Estimated data suggests that 74.6% of previously untreated older and/or comorbid patients remained alive and progression-free after 3.5 years of fixed-duration Ibr-Ven treatment, compared to 24.8% of patients in the Clb-Obi cohort.

## 4. Immunomodulatory Agents

### 4.1. Immune-Checkpoint Inhibitors

CLL is a disease of the mature B-cells; however, recent reports indicate the role of T-cells in the disease pathogenesis and progression [79,80]. In CLL, T-cell exhaustion is mediated by upregulation of co-inhibitory signals such as programmed death-1 (PD1), lymphocyte activation gene-3 (LAG-3), cytotoxic T-lymphocyte-associated protein-4 (CTLA4), and T-cell immunoglobin-3 (TIM-3). These findings have led to the development of immune-checkpoint inhibitors for managing CLL treatment [81]. However, PD1 inhibitors used as single agents have failed to produce promising results in CLL [82]. The anti-PD1 mAb pembrolizumab has shown selective efficacy in CLL patients progressing to RT. Recent trials in RT have demonstrated that combining ibrutinib with anti-PD1 mAb nivolumab has produced considerable results with acceptable levels of toxicity [83]. Moreover, a triplet combination of umbralisib, ublituximab, and pembrolizumab has reported durable responses [84]. As evidenced by high response rates in previously untreated RT patients, the combination of the Bcl2-inhibitor venetoclax, next-generation anti-CD20 mAb obinutuzumab, and anti-PDL1 mAb atezolizumab offer immunotherapy as a promising treatment approach.

### 4.2. Bispecific Antibodies

Bispecific antibodies (bsAbs) are a promising approach that combines antibody therapies with cellular-mediated immunotherapy. BsAbs are antibodies with two binding sites directed at two different antigens or two different epitopes on the same antigen. There are two types of bsAbs: bispecific T-cell engagers (BiTEs) and dual-affinity targeting antibodies (DARTs). BiTEs are a subtype of bispecific antibodies that link two single-chain variable fragments with a flexible linker. One fragment binds to the tumor-associated antigen, and the other binds to a T-cell-specific antigen to activate the T-cell to kill the cancer cell to which it is linked. DART consists of two variable fragments that connect the opposite heavy chain variable regions through a disulfide bond, improving stability. Blinatumomab, a CD19/CD3 bsAb designed in a BiTE format, was one of the first bsAbs tested in CLL and has been shown to eliminate CLL cells in a mouse xenograft model [85]. Blinatumomab was tested in vitro on 28 freshly treated naïve CLL patients. The antibody treatment induced tumor cell death via T-cell activation and granzyme-mediated cytotoxicity [86]. Clinical trials currently underway involve combining lenalidomide (NCT02568553) or blinatumomab-expanded T-cells (NCT03823365) in patients with a broad spectrum of NHL, including CLL. Promising results have emerged from preclinical studies of another bsAb called MGD011 CD3 X CD19 DART, which has shown the ability to effectively engage CLL-derived T-cells and promote the killing of tumor cells in vitro. Further, MGD011 also indicated an impact on eliminating CLL resistance to venetoclax [87]. Recently, preclinical studies have explored the potential of a bispecific antibody that targets leukemic cells and Vγ9Vδ2 T-cells, a conserved T-cell subset with intrinsic anti-tumor activity. Furthermore, a CD40-bispecific γδ T-cell engager has been found to trigger apoptosis through a powerful Vγ9Vδ2 T-cell-dependent anti-leukemic response [88]. Altogether, bsAbs may be a potentially valid option for high-risk patients resistant to previous therapies.

### 4.3. Bi- or Tri-Specific Killer Engagers

Natural killer cells are hypofunctional in CLL, affecting target cell recognition and cellular toxicity [81]. BiKEs (Bi-specific Killer Engagers) and TriKEs (Tri-specific Killer Engagers) recruit NK cells to target tumor antigens. The NKG2D receptor-ligand ULBP2 has been targeted by TriKEs (ULBP2/aCD19/aCD19 and ULBP2/aCD19/aCD33) and has demonstrated in vitro and in vivo activity against CLL [89]. Further, a CD16/CD19 BiKE and a CD16/CD19/CD22 TriKE have been shown to trigger NK cell activation via CD16 signaling, for which CD16/CD19 TriKE induced better killing of CLL cells in vitro compared to rituximab [90]. Overall, inducing an NK cell response against CLL cells is compelling to explore as a therapeutic option.

### 4.4. CAR T-Cells

Chimeric antigen receptor T-cells (CAR T-cells) represent a promising area of investigation in adoptive cellular therapies, combining the strengths of T-cells and antibodies to boost T-cell anti-tumor activity. To date, there have been four generations of CAR T constructs developed. These constructs typically consist of an antigen binding domain, such as a single chain variable fragment (scFv) derived from immunoglobulin directed against the tumor antigen, as well as the intracellular domain from the CD3 chain, and a costimulatory domain, which is generally identified as the intracellular domain of a costimulatory molecule (CD28/4-1BB) [91]. One of the key advantages of CARs is their ability to identify and target tumor antigens in an HLA-independent manner, thus targeting tumor cells in a tumor-evasive environment [92].

CD19 CAR T-cells have been widely used in B-cell malignancies; however, their usage in CLL is controversial due to exhausted T-cell phenotype in CLL [93,94] and the loss of CD19 upon CAR T therapy resistance. In the CLL 4 trial, CD19 CAR T-cells were used as a single agent, resulting in an ORR of 82%, with a 45% CR and a high rate of uMRD observed in heavily pre-treated CLL patients, including high-risk patients who were refractory to BTKi and venetoclax [95]. Other studies of CD19 CAR T-cells in CLL have also shown an ORR of 50–70% and a CR of 20–30% [96,97]. Kappa or lambda light chains can be attractive targets for CLL patients, as it allows high target specificity for the leukemia cells while avoiding the normal B-cells. Recently, a new CAR T has been investigated against the Ig light chain, as CLL cells mainly express the Ig light chain compared to their normal counterparts [98]. Preclinical studies showed CAR T-cells have been effective against Igκ or Igλ in vitro or in vivo CLL models, and there is an ongoing clinical trial investigating anti-Ig kappa on CLL (NCT04223765). Combination therapies have also been tested. A recent study suggested increased viability and expansion in human CAR T-cells in the presence of ibrutinib. In line with this, administering ibrutinib with anti-CD19 CAR T has increased tolerability with a lower incidence of severe side effects. One of the impediments to CAR T treatment in CLL is decreased fitness and activity of CAR T-cells due to immune subversion. Thus, using allogenic CAR T-cells from healthy donors has been an attractive option; however, strategies are evolving, such as gene editing-based strategies to knock out endogenous αβTCR to prevent graft-versus-host disease and donor-mediated rejection [99].

Similarly, acalabrutinib has been shown to improve the in vitro and in vivo anti-tumor function of CD19 CAR T-cells [100]. These studies’ results will guide future treatment when including CAR T-cells. Even though CD19 as an antigen has been a promising target, there are resistance mechanisms, such as a CD19 loss, leading to exploration for novel targets. Another good target is CD20. Anti-CD20 CAR has been investigated in non-Hodgkin lymphomas. Among the three patients who received anti-CD20 CAR, two did not develop the evaluable disease with a progression-free survival of 12 and 24 months. The third patient achieved partial remission and relapsed after 12 months post infusions [101]. An ongoing trial evaluates anti-CD20 CAR in R/R B-cell malignancies, including CLL (NCT0327779). Another attractive antigen expressed on CLL cells but not on normal B-cells is the ROR1 (Receptor tyrosine kinase-like orphan receptor 1) receptor [102]. In vitro, promising data shows CAR T’s effect on ROR1 and an ongoing trial evaluating anti-ROR1 CART against ROR1 malignancy, including CLL (NCT02706392). FcμR is expressed highly in CLL cells while at minor levels in normal healthy B-cells. Anti-FcμR CAR T has been investigated in CLL cells, which affects CLL cells without affecting normal healthy B-cells [103].

## 5. Emerging Novel Targets for CLL Treatment

### 5.1. Targeting RNA Splicing Dysregulation in CLL

Splicing is a highly precise and stepwise process that converts pre-mRNA into mature RNA, facilitated by a group of proteins called the spliceosome. The spliceosome is composed of over 300 proteins, which includes more than 100 accessory proteins that process the U2-type introns. The core of the spliceosome includes U1, U2, U4, U5, and U6 small nuclear ribonucleoproteins (snRNPs), as well as seven Sm proteins or Lsm (U6-specific) proteins and other snRNP-specific factors [104]. Each snRNP contains a small nuclear RNA (snRNA), enabling interactions between RNA–RNA and RNA–protein during the dynamic splicing process [104,105]. Typically, cells generate various mRNA forms via alternative splicing, which occurs through multiple mechanisms such as alternative 5′ or 3′ splice sites, exon skipping, alternative promoter, intron retention, and alternative polyadenylation [106].

Genome-wide cancer sequencing studies have identified recurrent mutations in RNA splicing factor proteins (*SF3B1*, *U1 snRNA*, *SRSF2*, *U2AF1*, *ZRSR2)* myeloid neoplasms, clonal hematopoiesis, mantle cell lymphoma, and CLL [11,15,107,108,109,110,111,112]. All these mutations lead to transcriptome-wide RNA splicing dysregulation. Additionally, RNA sequencing studies of primary cancer cells across various cancer types have revealed that aberrant RNA splicing is a common feature of cancer. In TCGA (The Cancer Genome Atlas) analysis of 33 different cancer types, mutations in 119 splicing factors were reported, which comprise half of the splicing factor proteins [113]. Moreover, 70% of splicing factors and 84% of RNA binding proteins are dysregulated at mRNA levels in various cancers, resulting in dysregulated splicing events.

The discovery of splicing factor mutations has generated an interest in therapeutic targeting of the splicing factor mutant tumor cells. One interesting feature in splicing factor mutant cases is the solid mutual exclusivity. Several reports also suggest that co-expression of the most common splicing factor mutations in *SF3B1*, *SRSF2*, or *U2AF1* is not tolerated in cells [114]. Similarly, expression of a single wild type encoding these factors is tolerated, while deletion of the wild type in splicing factor mutant cell lines leads to cell death [115]. This evidence further motivated the development of inhibitors to target the splicing catalysis function to kill splicing factor mutant tumors. Among the various inhibitors developed are a class of natural products and their synthetic analogs that bind to the Sf3b complex and prevent interaction with the branch point. Among the widely studied compounds, the pladienolide analogs (A-G and synthetic analog E7107) target Sf3b, which binds the U2 snRNP to disrupt splicing [116]. PLAD-B and FD-895 have been shown to induce apoptosis and overcome the protective effect of the microenvironment in CLL in vitro, indicating that these inhibitors may work in R/R CLL patients. Another pladienolide analog, E7107, has been tested in phase I clinical trials and showed limited efficacy; however, due to adverse events, the trial was terminated [117]. A recent report suggests the combination of E7107 with venetoclax sensitized both human and murine CLL cells in vitro and can overcome venetoclax resistance in vivo [16]. Another class of inhibitors similar to pladienolides is FR901464 and its methylated derivative spliceostatin A (SSA), which inhibit the Sf3b subcomplex [118]. Bcl2 family member Mcl1, an apoptosis regulator, is highly expressed in CLL samples with progressive disease. Reports suggest that spliceosome inhibitor spliceostatin (SSA) altered the splicing of Mcl1 and led to the downregulation of Mcl1, resulting in apoptosis [119]. Notably, the microenvironmental signals such as CD40L and IL4 treatment of CLL cells offered resistance to spliceostatin. This resistance was reversed using a combination of spliceostatin with ABT-199/263-BCL2 family inhibitor, indicating that the combination may work for CLL cells resistant to spliceostatin [119]. Another synthetic analog of FR901464, sudemycin, has been shown to induce apoptosis in CLL samples without affecting the normal B-cell counterparts in vitro and in vivo [120]. Further, combining sudemycin with ibrutinib confers enhanced sensitivity to ibrutinib by modulating the loss of regulatory function of IBTK over BTK via alternative splicing regulation [120]. Despite the promising results with splicing inhibitors in CLL, none of the inhibitors have been approved by the FDA for treatment, hence further understanding is needed to modulate splicing catalysis in vivo with an acceptable therapeutic index.

### 5.2. Targeting Metabolism in CLL

Metabolic changes enable the tumor cells to sustain proliferation and adapt to stressful conditions. Hanahan and Weinberg noted that metabolic rewiring is a hallmark of cancer cells [121]. Even though CLL cells are known to be quiescent, they have been shown to have high mitochondrial respiration and reactive oxygen species and enhanced antioxidant activity compared to normal B-cells [17,122,123]. Accumulating evidence indicates that CLL cells undergo spontaneous apoptosis, and a gradual increase in the size of the CLL clone results from the newly proliferating lymphocytes. CLL cells are generally slowly proliferating, with approximately 0.1% to 1.75% of CLL cells proliferating daily compared to resting B-cells [123,124]. Very few studies are investigating the role of metabolism in CLL and exploiting it as a therapeutic approach. We will briefly discuss the recent works related to metabolism in CLL.

#### 5.2.1. Mitochondrial Metabolism

**Figure 2:**
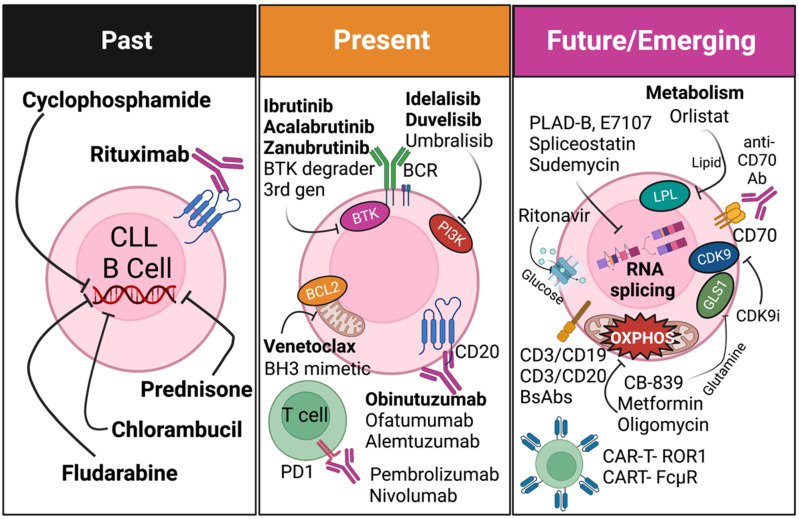
Summary of past, present, and emerging treatments in CLL. Created with Biorender.com.

Mitochondria play an important role in energy metabolism, as they regulate oxidative phosphorylation, reactive oxygen species, and ATP production via the TCA cycle. Mitochondria also regulate other metabolic processes, such as amino acid and fatty acid metabolism. CLL cells have higher mitochondrial mass, ROS, and activity than normal B-cells [18,19,123]. Primary CLL samples have been shown to have high basal respiration via seahorse-based assays [19]. Recent omics analyses indicate that CLL proliferation is linked to the mTOR-MYC-OXPHOS pathway [122]. Overexpression of oxoglutarate dehydrogenase and isocitrate dehydrogenase was commonly found in CLL cells [125]. In line with this, CLL cells are sensitive to the pharmacological inhibition of oxidative phosphorylation by OXPHOS inhibitors (PK11195, oligomycin A, and metformin) (Figure 2) [126].

#### 5.2.2. Glucose Metabolism

Glucose can be converted to pyruvate via glycolysis and ribose via the pentose phosphate pathway. Glucose is further utilized to synthesize glycogen, fatty acid, and serine. An increase in glycolytic flux to produce ATP meets the energy demands of highly proliferating cells, rendering the cells addicted to glucose. CLL cells have been shown to have high glucose metabolism and uptake. Glucose uptake inhibitors (ritonavir) and glycolysis inhibitors (2-DG) were reported to induce in vitro cytotoxicity in CLL [127]. *ATM* and *TP53* have been shown to regulate central carbon metabolism. *ATM* deletion or 11q deletion in CLL cells led to increased insulin receptor expression and glucose uptake [128]. Given this evidence, 11q-deleted CLL cells are more sensitive to glycolysis inhibition. Currently, there are clinical trials targeting the mitochondrial OXPHOS via metformin (NCT01750567) alone or in combination with GLUT4 (glucose transporter) via ritonavir (NCT02948283) [126].

#### 5.2.3. Glutamine Metabolism

Glutamine is the most abundant non-essential amino acid in the blood at a concentration of ~0.5–1 mM. Even though cells can synthesize glutamine via GLUL, many tumors are addicted to glutamine, especially KRAS and Myc-driven tumors [129,130]. Glutamine is converted to glutamate by a rate-limiting step in glutamine catabolism via glutaminase (GLS1). Glutaminase is overexpressed in CLL samples, targeted in vitro by CB-839. Del11q CLL patients exhibit higher glutamine synthesis and metabolism than their negative counterparts. Del11q CLL samples are susceptible to glutaminase inhibitors indicating the pivotal role of glutamine metabolism in Del11q CLL [126]. Glutamine is taken up by the cells via glutamine transporters such as SLC1A5, SLC38A1, and SLC38A2 [131]. CLL cell proliferation is inhibited by targeting the glutamine transporters via the V9302 inhibitor, indicating the vital role of glutamine transport in CLL [17]. To evaluate glutamine incorporation in CLL cells, there is an ongoing clinical trial (NCT04785989) in low disease burden CLL testing the glutamine incorporation via in vivo labeling.

#### 5.2.4. Lipid Metabolism

Metabolomic analysis of CLL samples shows a differential abundance of lipids compared to other metabolites in CLL, indicating the dependency of CLL on fatty acid metabolism [18,124]. Lipoprotein lipase mRNA levels are highly expressed in CLL compared to normal B-cells [132]. BCR stimulation further increases the LPL expression, indicating the function of BCR signaling in regulating fatty acid metabolism in CLL. Fatty acid synthesis and oxidation genes overexpressed in CLL are reported [133]. Orlistat, a fatty acid synthesis inhibitor, is cytotoxic for CLL cells in vitro. CPT-1, a mitochondrial fatty acid transporter, is highly expressed in CLL [134]. In line with this, CLL cells are sensitive to etomoxir, which targets fatty acid oxidation via CPT-1. Recent reports also suggest that ibrutinib affects fatty acid metabolism via inhibiting free fatty acid synthesis [135]; however, the mechanism is poorly understood.

## 6. Future Therapies

### 6.1. Combination Therapies

#### 6.1.1. Targeted Therapy Coupled with Other Novel Chemotherapy

Based on extensive CIT studies, in 2016 the European Medicine Agency accepted the use of uMRD, defined as <1 CLL cell per 10,000 leukocytes, as an intermediate endpoint and independent prognostic factor for PFS and OS [136,137,138]. However, uMRD and CR are not commonly achieved with targeted agents such as ibrutinib, and several combinations are being tried to achieve increased efficacy. The HELIOS trial involved 578 R/R CLL patients who were randomly assigned to receive BR plus ibrutinib or BR plus a placebo. The results showed that adding ibrutinib led to a significantly higher ORR (83 vs. 68%), longer median PFS, and a higher rate of MRD negativity (13% vs. 5%). In total, ibrutinib improved the efficacy of the treatment in both naïve patients with del(17p) and R/R patients compared to CIT treatment [139,140]. However, some off-target ibrutinib may be responsible for its unique toxicities, such as atrial fibrillation and bleeding.

Similarly, PI3Kδ idelalisib has been tested with BR. The triple combination of PI3K with BR produced a significant increase in PFS (20.8 vs. 11.1 months) compared to BR alone; however, the triple combination was associated with adverse events such as increased infections, limiting its clinical use [141]. Another trial tested the combination of ibrutinib with FC and obi in young treatment-naïve patients with a favorable genetic profile (IGHV mutated and no TP53 aberrations). MRD was used to guide frontline therapy [142]. The patients in the study received a quadruple combination for three courses, followed by either ibr plus obi for nine cycles or ibr plus obi for three cycles and ibrutinib for six cycles, based on their MRD status post-chemoimmunotherapy. Of the 28 patients who completed the 12-month treatment, all achieved undetectable MRD and a CR rate of 86%. Though these kinase inhibitors achieve impressive undetectable MRDs and CR, treating elderly patients with comorbidities becomes difficult due to increased toxicity rates.

#### 6.1.2. Novel Agents in Combination with Anti-CD20 Antibodies

The ALLIANCE randomized phase III trial compared ibrutinib and ibrutinib plus rituximab to bendamustine plus rituximab in older patients and found the efficacy to be almost identical for both the ibrutinib-containing arms [143]. In the iLLUMINATE trial, Ibr-Obi was compared to Chl-Obi, with the former achieving superior PFS at 30 months (79% vs. 31% *p* < 0.0001). In the relapsed/refractory R/R setting, rituximab plus venetoclax, not ibrutinib, has produced improved MRD-negative rates, leading to its broad approval in the R/R setting [144,145]. In the CLL2-BAG trial, a combination of bendamustine, venetoclax, and obinutuzumab was used, and an MRD-guided maintenance phase led to an 87% rate of MRD negativity [41,45]. CLARITY is a phase II trial in which a combination of ibrutinib plus venetoclax was tested in 40 patients with R/R CLL. The results showed a CR rate of 58% (23/40) and no detectable MRD in peripheral blood after 12 months of treatment. In another trial of treatment-naïve CLL patients (*n* = 80), the same combination was tested for 24 months. Ninety-six percent of patients treated with a combination of venetoclax and ibrutinib achieved a complete response (CR) after 12 months, and 69% had no undetectable MRD in the bone marrow [146]. Combined treatment can achieve better efficacies; however, optimal treatment selection for each patient is challenging.

#### 6.1.3. Targeted Therapy Coupled with an Immunomodulatory Agent

Lenalidomide has been used as an immunomodulatory agent, which has been shown to induce T-cell activation resulting in CLL cell apoptosis. A combination of lenalidomide with idelalisib can potentially reduce lenalidomide-induced flare, as lenalidomide-induced cytokine release and immune activation are PI3K dependent [147]. The most common toxicities are fatigue, thrombocytopenia, and neutropenia, noted in 83%, 78%, and 78% of patients, respectively [148]. The ORR in different studies has been 32–54% with monotherapy and is better (66%) in combination with rituximab. In another trial, lenalidomide was evaluated for maintenance therapy post-chemotherapy in high-risk patients (NCT01556776).

### 6.2. Allogenic Transplant

Allo-SCT has been considered an option for high-risk CLL patients in the CIT era and remains one of the potentially curative treatments for CLL. In this era of novel agents, allo-SCT remains an option for high-risk patients who progress after at least BTKi or venetoclax treatment. Due to substantial toxicities and morbidities, myeloablative-based treatment strategies were discontinued in CLL patients. However, recently large-scale prospective studies conducted with a median follow-up of 6 years [149,150,151] have shown that RIC (reduced-intensity conditioning) allo-SCT can provide long-term disease control in 40% of patients and overcome TP53-based negative prognostication and refractoriness associated with fludarabine. In different studies, OS has been 50% with a PFS of ~40% [152]. Non-relapse mortality (NRM) remains significant, affecting 15–25% of patients. In a prospective trial conducted on 55 patients, adding rituximab peri-transplant improved response rates compared to 157 historical control patients. The NRM rate in patients with no comorbidities was less than 12% [153,154]. Limited data is available on allo-SCT efficacy; hence, more collaborative efforts are needed to understand the effectiveness of novel agents in this era.

### 6.3. Recent Targets and Trials in CLL Discussed in ASH2022

In the last year’s ASH, several new targets were discussed. As patients treated with Ibr-Ven show resistance, these authors explored Protac-based BCL2/BCL-XL degrader PZ18753b to overcome Ibr-Ven resistance. The authors used OSU-CLL cells to generate BCL2 mutant (G101V, F104, and R107-110dup) cells. They showed rapid apoptosis with Protac-based degrader PZ118753b with efficient degradation of BCL-xL and partial degradation of BCL2, suggesting that venetoclax resistant cells retain dependency on BCL2/BCL-xL [155]. As CDK9 is a master transcription regulator and a potential target in hematologic malignancies, the authors explored PRT2527 as a novel low nanomolar potent CDK9 inhibitor in B-ALL and CLL primary samples. Their studies showed that PRT2527 treatment has potent anti-leukemic activity in primary CLL and B-ALL samples evaluated ex vivo and two systemic models of B-ALL in vivo [156]. Another group explored bromodomain-containing protein 9 (BRD9), a chromatin remodeling complex, as a potential target in CLL. The authors showed that BRD9i could improve the survival of orthotopic CLL xenograft mice with significantly reduced expression of targets related to the NRF2 pathway [157]. CD70, a TNF family member, and its receptor CD27 were highly expressed in CLL cells compared to normal B-cells, suggesting immune deregulation in CLL. Using preclinical models, the authors showed that targeting CD70 with anti-CD70 antibodies in CLL altered BCR, CD40L, IL4, and TNFR signaling and delayed CLL disease progression, thus suggesting the use of anti-CD70 immunotherapy in combination therapies in CLL [158]. As RT studies need more bona fide models, from our group, we reported the establishment of a new RT murine model driven by *Mga* KO and OXPHOS, where targeting the OXPHOS regulation along with CDK9i could improve RT mice survival [159]. As RT has an unmet clinical need, the Phase Ib/II EPCORE CLL-1 trial explored Epcoritamab, a novel subcutaneously (SC) administered CD3/CD20 bispecific antibody in CD20+ RS LBCL (large B-cell lymphoma). Among the ten patients who received treatment, preliminary findings show that SC-Epcoritamab has encouraging single-agent activity with high overall and complete response rates. Most were seen at the first six-week assessment [160]. This is an ongoing trial; updated data will be presented in future studies.

## 7. Conclusions

The treatment of CLL has been transformed with the increasing availability of innovative agents that are now favored over traditional CIT in all settings. However, there are still many questions to be answered through well-designed studies. Areas of active research include RT metabolism, new targets, combinational treatments in CLL, splicing-related inhibitors, and the best treatment approach for high-risk patients. The cost-effectiveness of different treatments must also be taken into account. Although there are many obstacles, CLL therapy is still hopeful, with new agents recently approved by the FDA and more promising ones coming. Based on the ongoing recently completed accrual from several cooperative groups and international phase III trials (EA9161: NCT03701282; A041702: NCT03737981; CLL17: NCT04608318; MAJIC: NCT05057494), the proposed treatment can include treatment arms combining venetoclax and BTKi in the frontline setting. A viable strategy for relapsed or refractory CLL patients without identified BTK/PLCG2/BCL2 mutations may involve retreatment with either ibrutinib/non-covalent BTKi, venetoclax, or both. Lastly, advances in disease biology have revealed new targets and brought us closer to a cure for CLL by shifting from chemotherapy to more patient-friendly treatments.
